# Human H5N1 influenza infections in Cambodia 2005–2011: case series and cost-of-illness

**DOI:** 10.1186/1471-2458-13-549

**Published:** 2013-06-06

**Authors:** Karen Humphries-Waa, Tom Drake, Anthony Huszar, Marco Liverani, Khieu Borin, Sok Touch, Teng Srey, Richard Coker

**Affiliations:** 1 Communicable Disease Policy Research Group, London School of Hygiene & Tropical Medicine, Faculty of Public Health, 9th Fl. Satharanasukwisit Building, 420/1 Rajvithi Road, Bangkok 10400 Thailand; 2 CelAgrid, PO Box 2423, Phnom Penh 3, Cambodia; 3 Communicable Disease Control Department, Ministry of Health, Kampuchea Krom Blvd, Phnom Penh 151-153, Cambodia

**Keywords:** Avian, Influenza, H5N1, Cambodia, Cost-of-illness

## Abstract

**Background:**

Southeast Asia has been identified as a potential epicentre of emerging diseases with pandemic capacity, including highly pathogenic influenza. Cambodia in particular has the potential for high rates of avoidable deaths from pandemic influenza due to large gaps in health system resources. This study seeks to better understand the course and cost-of-illness for cases of highly pathogenic avian influenza in Cambodia.

**Methods:**

We studied the 18 laboratory-confirmed cases of avian influenza subtype H5N1 identified in Cambodia between January 2005 and August 2011. Medical records for all patients were reviewed to extract information on patient characteristics, travel to hospital, time to admission, diagnostic testing, treatment and disease outcomes. Further data related to costs was collected through interviews with key informants at district and provincial hospitals, the Ministry of Health and non-governmental organisations. An ingredient-based approach was used to estimate the total economic cost for each study patient. Costing was conducted from a societal perspective and included both financial and opportunity costs to the patient or carer. Sensitivity analysis was undertaken to evaluate potential change or variation in the cost-of-illness.

**Results:**

Of the 18 patients studied, 11 (61%) were under the age of 18 years. The majority of patients (16, 89%) died, eight (44%) within 24 hours of hospital admission. There was an average delay of seven days between symptom onset and hospitalisation with patients travelling an average of 148 kilometres (8-476 km) to the admitting hospital. Five patients were treated with oseltamivir of whom two received the recommended dose. For the 16 patients who received all their treatment in Cambodia the average per patient cost of H5N1 influenza illness was US$300 of which 85.0% comprised direct medical provider costs, including diagnostic testing (41.2%), pharmaceuticals (28.4%), hospitalisation (10.4%), oxygen (4.4%) and outpatient consultations (0.6%). Patient or family costs were US$45 per patient (15.0%) of total economic cost.

**Conclusion:**

Cases of avian influenza in Cambodia were characterised by delays in hospitalisation, deficiencies in some aspects of treatment and a high fatality rate. The costs associated with medical care, particularly diagnostic testing and pharmaceutical therapy, were major contributors to the relatively high cost-of-illness.

## Background

A future influenza pandemic has the potential to cause millions of deaths and significantly impact on the global economy [1]. Southeast Asia has been identified by the World Health Organisation (WHO) as being at risk for emerging diseases including avian influenza [2]. There have been a number of cases of highly pathogenic avian influenza (HPAI) subtype H5N1 in the region which has had a serious impact on the poultry industry and associated livelihoods [3,4]. Whilst mortality in domestic poultry has been very high, it is the potential to cause serious disease in humans and the subsequent risk of pandemic spread that causes particular concern [4]. For many patients, HPAI infections follow an unusually aggressive clinical course, with rapid deterioration and high fatality [4,5]. Between 2003 and 2011, 574 human cases of H5N1 were reported worldwide [6]. Of these, 60% of the cases and 72% of the deaths have been in the Southeast Asia [6].

A number of factors contribute to Southeast Asia’s vulnerability to infectious diseases including population growth and movement, urbanisation, environmental factors such as agriculture, land use, water and sanitation, health system factors and the development of drug resistance [3]. The close contact between animals and humans, particularly in rural settings also enhances the vulnerability for outbreaks of zoonotic infections such as HPAI [3]. Countries with the lowest income will suffer the greatest burden from disease [3]. Cambodia is one of the poorest countries in Southeast Asia ranking 138^th^ on the United Nations Development Program Human Development Index [7]. Data from the WHO Burden of Disease, confirms the burden of communicable disease weighs more heavily on Cambodia than other countries within the region [8].

The WHO has expressed concern regarding the inadequacy of global preparedness for an influenza pandemic [3]. Cambodia, like many other developing countries, has a limited and likely insufficient level of pandemic preparedness to deal with an outbreak of a highly pathogenic and transmissible strain of influenza [3,9-12]. Based on data collected regarding key health system resources, Cambodia has been identified as having some of the largest resource gaps and potentially the highest rate of avoidable deaths when compared to other countries in Southeast Asia, including Indonesia, Lao PDR, Taiwan, Thailand and Vietnam [12]. Cambodia has the lowest density of health professionals per capita in the region; insufficient healthcare facilities with no surge capacity; equipment shortages including mechanical ventilators; and only 0.025% of the WHO recommended national stock of anti-viral tablets [3,9-12]; Sok Touch, Ministry of Health Cambodia, personal communication]. Additionally Cambodia does not have any discretionary budget for local level administration for influenza or pandemic preparation [10]. To address these issues, the Royal Government of Cambodia has developed a comprehensive multi-sectoral pandemic preparedness plan [13].

An influenza pandemic may not only result in significant loss of life but also have a substantial impact on the economy. The 2003 SARS outbreak demonstrated that even a disease with relatively limited health impacts can have a major effect on the economy of a region [14]. The Asian Development Bank estimate the impact of SARS in East Asia was approximately US$18 billion and suggest that an influenza pandemic could be substantially more [14]. Compared to SARS, HPAI has not significantly impacted tourism however it has been extremely damaging to the poultry industry [3,14]. The cost to the poultry sector in Cambodia, Thailand and Vietnam has been estimated at US$560 million [14]. Smallholder farmers dependent on poultry production are more likely to have difficulty in overcoming the costs of culling and restocking [15]. Backyard systems of poultry farming have traditionally dominated the industry in Cambodia with close proximity of fowl in villages or backyards, minimal biosecurity and local consumption [15]. Those living or working closely with poultry are more at risk of contracting HPAI and incurring illness-related costs. Unfortunately it is the poor who are particularly at risk of the zoonoses associated with livestock keeping; they are also those least able to afford the cost-of-illness (COI), the resources expended or foregone as a result of disease [3,16]. There has been no research published regarding the COI for HPAI in the Southeast Asia. This study seeks to determine the COI in Cambodia, one of the most vulnerable countries in that region.

At the end of 2011, eighteen laboratory-confirmed cases of H5N1 had been reported in Cambodia [6]. Here we present details of these cases, estimate the COI and compare these findings, where possible, with others reported from the region. We do so with the aim of better understanding the course of HPAI in Cambodia and to provide a foundation on which to develop recommendations for policies and plans to facilitate pandemic mitigation.

## Methods

All cases included in this study were identified between January 2005 and August 2011 and confirmed as positive for the H5N1 virus using the polymerase chain reaction (PCR) test by the laboratories of the Institut Pasteur, in either Phnom Penh or Ho Chi Minh. These cases had then been reported to the Centre for Communicable Diseases in the Cambodian Ministry of Health (MOH), Phnom Penh. Data for these patients was collected, with permission from the MOH, through review of medical records and interviews with staff at the admitting hospitals during a field visit in December 2011. A general study consent form was utilised for in-depth interviews. Verbal assent was used in place of written consent for information gathered in the hospital setting from shorter, less formal interactions.

This study takes a societal perspective regarding the cost-of-illness for HPAI, meaning indirect costs to patients and their families are included in addition to medical provider costs [17]. This approach was taken in order to determine the full economic cost of illness. Data collection utilised an ingredients approach with micro-costing of key elements [17]. Costs were gathered in or converted, where necessary, to US dollars [18]. Whilst all reported patients originated in Cambodia and were reported to the Cambodian MOH, two patients were hospitalised in Vietnam. These cases were excluded from the cost-of-illness analysis since data, regarding some aspects of care and the associated costs, was not available. Adjustments were made by inflation or deflation to 2011 parity using inflation rates cited by the International Monetary Fund and the Cambodian National Institute of Statistics [19,20]. The medical provider cost centres used in analysis were outpatient consultations, inpatient facility costs including capital and personnel costs, pharmaceuticals, diagnostic tests and oxygen administration. Diagnostic tests included viral PCR, anti-viral phenotypic testing, full blood counts, renal function tests (urea, creatinine, and other electrolytes), liver function tests, acute phase proteins as well as urine dipstick tests. The costs of full genomic sequencing of the viral strain and tests to detect antigenic drift were not included as these were assumed to be research related. The pharmaceuticals utilised were categorised as antivirals, antibiotics, antipyretics, corticosteroids or other medication. The costs centres attributed to the patient or family included travel expenses to the hospital of admission, food whilst hospitalised and the indirect cost of loss of income for the patient or caregiver due to hospitalisation. Information on treatment costs recovered from patients or organisations paying user fees on behalf of the patients was not available.

WHO country specific unit costs, adjusted for inflation, were utilised in accounting for outpatient consultations at rural health services, public health centres, physicians’ clinics and nurse visits [21]. Other costs were determined from data gathered in April-June 2012 from the Institut Pasteur, Ministry of Health, retail pharmacies and interviews with physicians and staff at hospitals in Kampot and Angkor Chey. In general, outpatient consultations and hospitalisation are represented by actual costs where as charges are utilised for pharmaceuticals and diagnostic tests due to the lack of cost data at the time of writing. Retail pharmacy pricing was utilised when data was unavailable from hospital visits. A recent study, commissioned by University Research Company assessing hospital costing and financial management, provided hospitalisation costs per bed day, excluding in kind drugs [22]. The values reported for bed per day are very similar to those cited by WHO data (adjusted for inflation) with a hospitalisation cost per day of $8 to $10 [22] compared to $7 to $9 [21]. Travel costs to the admitting hospital were derived using the University Research Co. model which takes into account not only distance travelled but also the quality of the roads [T Jordanwood, University Research Co., LLC, Cambodia, personal communication]. With regard to lost income, this was calculated using a human-capital approach as a combination of both patient and caregiver earnings. It was assumed, based on employment statistics from the Cambodian National Institute of Statistics (NIS), that 74% of all caregivers and patients over the age of 18 years would be employed but only 20% of those aged between 6–18 years and no children under the age of six years [20]. It was also assumed that all children under the age of 6 years required the presence of a full-time caregiver; 50% of those aged 6–18 years had a caregiver present; and that no caregiver was present for those over the age of 18 years. Average income was based on NIS data and adjusted for inflation to US$237 per month, for a six day working week [20].

A multi-variate sensitivity analysis was performed using the average cost of the case series as the baseline cost-of-illness. Upper and lower scenarios were developed for the patient care in order to determine the impact on the COI cost centres. The minimal care comparator was outpatient care only, including a single consultation and the average cost of outpatient medication whilst the maximum utilised the full battery of diagnostics and treatment, including a complete adult course of oseltamivir and higher cost hospitalisation in an intensive care unit [22].

Ethics approval was obtained from the Cambodian National Ethics Committee for Health Research and London School of Hygiene and Tropical Medicine.

## Results

Six hospitals reported laboratory confirmed cases of H5N1 originating in Cambodia: the Kantha Bopha Children’s Hospitals in Phnom Penh and Siem Reap (six and four patients respectively), the Calmette Hospital in Phnom Penh (four patients), the Kieng Gieng Hospital in Vietnam (two patients), the Kampong Cham Regional Hospital (one patient), and the ‘Cambodia-Japan Friendship’ Mongkul Borie Provincial Hospital in Banteay Meanchey Province (one patient). The median age of those reported with H5N1 was 10.5 years (mean: 14.7 years, range: 11 months - 57 years) with the majority (11 of 18 patients, 61%) being under the age of 18 years. Twelve of the patients (67%) were female. There was a median delay of 7 days (mean: 7.3 days, range: 3–26 days) between symptom onset and hospitalisation and six days (mean: 6.2 days, range: 2 - 13 days) between initial outpatient treatment and viral testing. Sampling for the PCR test used in the diagnosis of the H5N1 virus was performed by the admitting hospitals with analysis carried out by the Institut Pasteur. Five patients were treated with oseltamivir of whom only two adults received the recommended dose of two capsules per day for five days. The remaining three child patients died before they could receive a full course of the anti-viral. Time from onset of symptoms to treatment with oseltamivir was a median of 9 days (mean: 12.0 days, range: 5 - 26 days). Children five years and younger were admitted a mean 5.7 days following symptom onset, whilst adults 18 years and older were hospitalised after 9.7 days. No patients received their first dose of oseltamivir within the recommended 48 hours from the onset of symptoms.

Many patients, travelled substantial distances to be admitted into hospital (mean distance of travel 167 km, range 8-476 km). Only two patients, those hospitalised in Vietnam, received invasive ventilation. An average of four antibiotics (range: 1 - 7) were used in the treatment of patients, with one third of patients receiving five or more. Ten patients (56%) received corticosteroid treatment. Sixteen of the 18 patients died giving a particularly high case fatality rate of 89%. Eight of the 16 patients, for whom data is available, died within 24 hours of admission to hospital. The most common cause of death was multi-organ failure or acute respiratory distress syndrome, precipitated by pneumonia and sepsis. The two surviving patients both received a full course of oseltamivir and were aged 19 years and 57 years.

### Cost-of-illness

**Figure 1:**
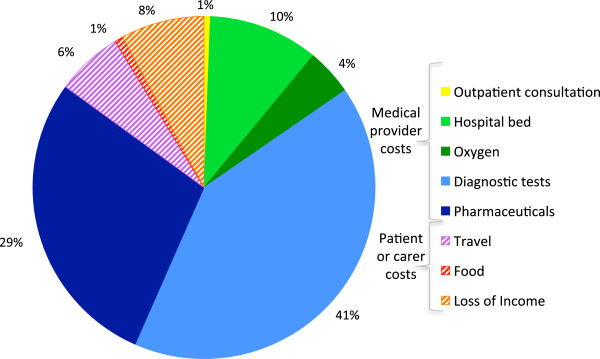
**The contribution of components to the cost-of-illness for H5N1 (% of total cost).** This pie chart illustrates the percentage contribution of the cost components to the total cost-of-illness for H5N1 HPAI in Cambodia. The cost components are divided into medical provider costs or patient and carer costs. Those costs, which are attributable to the patient or carer are cross-hatched.

The cost-of-illness was calculated for the 16 patients who received their care in Cambodia; the two patients who travelled to Vietnam for hospitalisation were excluded due to lack of data. The average cost-of-illness for H5N1 HPAI (2005 - 2011) was US$299.69 per patient of which 85.0% comprised medical provider costs. The components of the cost-of-illness are shown in Table 1. Of the medical costs the greatest contributor was diagnostic testing ($123.41, 41.2%) followed by pharmaceuticals ($85.15, 28.4%), hospital bed ($31.25, 10.4%), oxygen ($13.20, 4.4%) and outpatient consultations ($1.85, 0.6%). Of the diagnostic testing performed it was PCR viral testing that represented the most significant cost, being 32.2% of medical provider costs. Antibiotics and other medications made up most of the pharmaceutical costs being 13.0% and 14.3% of medical provider costs respectively whilst oseltamivir only contributed 1.3% due to low usage. Patient or family costs were estimated to be 15.0% of the cost of illness or approximately US$44.82 per patient. These costs increase however to US$55.77 or 18.6% if outpatient medical costs are included. Lost income and travel costs were the greatest contributors to family costs, representing $23.54 (52.5%) and $18.88 (42.1%) respectively (Figure 1).

**Table 1:** The cost-of-illness for patients diagnosed and hospitalised with H5N1 influenza in Cambodia

**Patient number**	**Medical provider costs (US$)**						**Patient and carer costs (US$)**				**Total cost (US$)**
	**Outpatient consultation**	**Hospital bed**	**Oxygen**	**Diagnostic tests**	**Pharmaceuticals**	**Total**	**Travel**	**Food**	**Loss of income**	**Total**	
1	1.41	30.00	11.74	135.47	112.56	291.62	16.00	2.93	19.48	38.41	**330.03**
2	2.82	10.00	3.91	113.43	119.50	250.09	16.00	0.73	8.25	24.98	**275.07**
3	1.41	40.00	15.65	109.67	355.16	522.41	12.00	3.91	25.97	41.88	**564.28**
4	1.41	40.00	15.65	116.65	57.41	231.64	38.00	3.91	25.97	67.88	**299.52**
5	1.41	8.00	3.91	82.15	37.18	132.92	40.00	0.49	6.49	46.98	**179.90**
6	1.41	80.00	31.30	124.53	129.92	368.03	24.00	5.86	65.97	95.83	**463.86**
7	1.41	10.00	3.91	101.08	33.69	150.35	8.00	0.98	6.49	15.47	**165.82**
8	1.41	10.00	3.91	125.18	19.74	160.51	26.00	0.49	6.49	32.98	**193.49**
9	1.41	72.00	35.21	88.18	30.61	228.37	6.00	4.40	58.43	68.82	**297.19**
10	2.82	80.00	39.12	183.17	109.64	415.98	14.00	4.89	64.92	83.80	**499.78**
11	2.82	20.00	7.82	149.89	50.04	231.09	14.00	1.47	16.49	31.96	**263.05**
12	1.41	40.00	15.65	115.69	115.01	288.28	18.00	3.91	25.97	47.88	**336.16**
13	2.82	20.00	7.82	82.15	48.99	162.31	32.00	1.47	16.49	49.96	**212.27**
14	1.41	20.00	7.82	164.20	63.62	257.40	10.00	1.95	12.98	24.94	**282.34**
15*	2.82										
16	1.41	10.00	3.91	131.21	0.05	146.84	14.00	0.49	6.49	20.98	**167.82**
17*	2.82										
18	2.82	10.00	3.91	151.95	79.32	248.43	14.00	0.73	8.25	22.98	**271.41**
**Mean**	**1.85**	**31.25**	**11.74**	**123.41**	**85.15**	**254.87**	**18.88**	**2.41**	**23.54**	**44.82**	**299.69**
**% of total costs**	**0.6**%	**10.4**%	**4.4**%	**41.2**%	**28.4**%	**85.0**%	**6.3**%	**0.8**%	**8.3**%	**15.0**%	**100**%

* Patients hospitalised in Vietnam were excluded from cost analysis.

**Figure 2:**
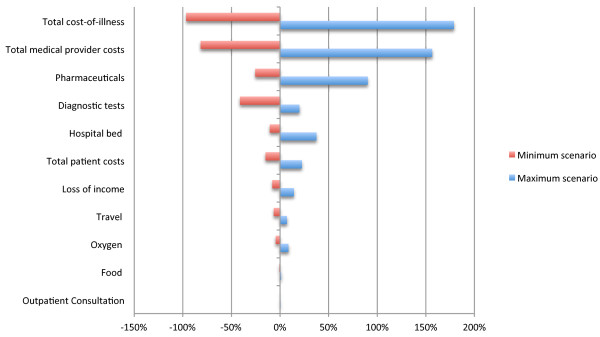
**Sensitivity of cost-of-illness for H5N1 to change in intensity of care (% change in total cost).** This tornado diagram was derived from a multi-way sensitivity analysis and illustrates the percentage change in total cost-of-illness when the intensity of care is varied. The baseline is the average COI from the case series. Upper and lower scenarios were developed for the patient care in order to determine the impact on the COI cost centres. The minimum care comparator incudes only outpatient consultation and loss of income due to illness whilst the maximum scenario utilises a full battery of diagnostics and treatment, including a complete adult course of oseltamivir and higher cost of hospitalisation in an intensive care unit.

For the two surviving patients the mean total COI was $397.63 whilst for the remaining patients, all of whom died, the COI was $285.70. Table 2 shows the results of the sensitivity analysis for cases receiving maximum and minimum care. The minimum care scenario, resulted in a total COI of $10.51, the majority being attributable to outpatient pharmaceuticals $9.10 (86.6%). The maximum cost scenario produced a total COI of $835.66, with the greatest contributors being pharmaceuticals (42.5%), diagnostic tests (21.9%) and hospitalisation (17.1%). The most notable impacts of a maximum care scenario is that of increased contribution to costs from hospitalisation in an intensive care unit and pharmaceuticals and the reduced relative input of diagnostic testing (Figure 2).

**Table 2:** Sensitivity analysis of cost-of-illness for patients diagnosed and hospitalised with H5N1 influenza

**Scenario**	**Medical provider costs US$ (% of row total cost)**						**Patient costs (% of row total cost)**				**Total cost US$ (% of average scenario)**
	**Outpatient consult**	**Hospital bed**	**Oxygen**	**Diagnostic tests**	**Pharma- ceuticals**	**Total**	**Travel**	**Food**	**Loss of income**	**Total**	
Minimum	1.41 (13.4%)	0.00	0.00	0.00	9.10 (86.6%)	**10.51** (100%)	0.00	0.00	0.00	**0.00**	**10.51 (4%)**
Average	1.85 (0.6%)	31.25 (10.4%)	13.20 (4.4%)	123.41 (41.2%)	85.15 (28.4%)	**254.87**(85.0%)	18.88 (6.3%)	2.41 (0.8%)	23.54 (7.9%)	**44.82 (15.0%)**	**299.69 (100%)**
Maximum	2.82 (0.3%)	143.30 (17.1%)	39.12 (4.7%)	183.17 (21.9%)	355.16 (42.5%)	**723.57** (86.6%)	40.00 (4.8%)	5.86 (0.7%)	66.22 (7.9%)	**112.09(13.4%)**	**835.66 (279%)**

## Discussion

The median delay between the onset of HPAI symptoms and hospitalisation of seven days was similar to that reported for other countries in the region, six days in Indonesia and Vietnam and five days in Thailand [23-25]. Children aged ≤5 years were hospitalised more quickly than adults although it still took an average of 5.7 days for children to be admitted. A study of 13 countries from Asia and the Middle East, also found more prompt hospitalisation of children ≤5 years [26]. Due to the reliance on hospitals to manage viral diagnostic testing, it was instigated a median of six days following initial treatment. Early identification of suspect cases is important for receiving prompt and appropriate medical care, which may improve outcomes and reduce the COI. Rapid immunoassay tests utilised at the community level may provide an opportunity to achieve more timely diagnoses. Notification of local poultry outbreaks may raise the suspicion of community health workers, directing viral testing and treatment with antivirals.

Fewer patients in Cambodia (28%) received antiviral treatment with oseltamivir, compared to those in Indonesia (69%), Thailand, (57-71%) and Vietnam (82%) and for those patients who did receive the drug, time to treatment was longer for the Cambodian group, (median nine versus seven days) [23-25]. This delay in antiviral treatment represents a significant deviation from the recommended 48 hours [4,5]. Whilst earlier treatment may be more beneficial, initiation and treatment with oseltamivir up to 6 - 8 days after symptom onset has been found to reduce mortality [5,26,27]. No parenteral or oral solution oseltamivir has been reported to be available in Cambodia and this may also have been a barrier to treatment of children. Problems procuring such formulations have been reported in other low-income countries, as have difficulties in adjusting doses for young children [27].

The excessive use of antibiotics in the treatment of HPAI is of concern considering the potential for the development of antibiotic resistance amongst bacterial strains. Corticosteroids were also commonly prescribed, despite a lack of evidence regarding effectiveness and the WHO recommendation against routine use in the treatment of H5N1 [27]. Finally none of the patients hospitalised in Cambodia received mechanical ventilation probably as a result of equipment shortages which have been reported in all provinces [12]. The WHO recommend investment in oxygen systems to improve the diagnosis and management of hypoxaemia as part of health system support for the clinical management of influenza [27]. Hypoxaemia is a common and major cause of mortality in those patients suffering pneumonia and a lack of mechanical ventilators is likely to contribute significantly to avoidable deaths [12,27].

The case fatality rate of 89% for patients diagnosed with HPAI in Cambodia to the end of 2011 is notably higher than the rate of 58.7% reported globally [6]. It is also higher than other case fatality rates (CFR) reported in Southeast Asia, (Vietnam 50%, Thailand 68% and Indonesia 82%,) with the exception of Laos where the only two reported cases have proved fatal [6]. Since data collection ceased, at the end of 2011, until April 2013 there have been 13 further cases of H5N1 diagnosed in Cambodia, with 11 fatalities, bringing the CFR to 87% [6]. The 100% CFR of those under the age of 18 years also contrasts with the findings of the study multi-country study by Oner et al. which found children to have a lower CFR than adults, 48.7% for those under 18 years and 28% for those ≤5 years [26]. The median duration between admission and death was shorter than that reported in Indonesia, (one compared to three days), with more patients dying on the day of admission (44% in Cambodia versus 12% in Indonesia) [23]. This contrasts with Thailand where fatal cases were hospitalised for a median of six days before death [24]. The death of the majority of patients within two days of hospitalisation suggests that, like those patients in Indonesia, the Cambodian cases had progressed to a stage when treatment was unlikely to impact on clinical outcome [23].

Delays in the initiation of medical care and antiviral therapy have been associated with increased severity of influenza and undoubtedly contributed to the high fatality rate of HPAI in Cambodia [26,27]. Barriers to accessing health services have been reported to include physical barriers such as distance, transport, waiting times; financial barriers including direct and indirect costs; quality of care; user knowledge and confidence; and sociocultural barriers, including restrictions due to family circumstances and reliance on traditional medicine [28]. The extent of the distance travelled, including in two cases border crossings to Vietnam, may reflect a distrust of public services and a desire to avoid the associated user charges whether official or under-the-table [28]. Indeed the Kantha Bopha Children’s Hospitals attended by 10 of the 18 patients (56%), is privately owned, funded by charitable donations and provides completely free care of an anecdotally high standard. Certainly patients or their caregivers were motivated to travel since travel expenditure represents a significant personal cost.

The mean cost-of-illness for HPAI in Cambodia amounted to $299.69 per patient, with the majority of this cost, 85.0%, attributable to the medical provider costs particularly those related to diagnostic testing (41.2%) and pharmaceutical management (28.4%). The cost of HPAI in Cambodia is notably higher than that which has been reported for dengue fever ($27 to $75 average cost per case) and that of hospitalised cases of dengue and other febrile illness ($36.20-$55.40) [29,30]. In the latter report the direct medical costs were a lesser contributor, being 33-35% of the COI, with the balance attributable to non-medical and indirect costs [29]. A notable factor in the cost difference between HPAI and dengue relates to the diagnostic testing and pharmaceutical management of HPAI. In a pandemic situation PCR viral tests and phenoytypic testing are unlikely to be feasible for all patients due to patient load and therefore clinical diagnosis is more likely. However laboratory diagnosis of HPAI may be critical to receiving appropriate treatment, including use of antiviral treatment. Improvements in the pharmaceutical management of HPAI including the prompt use of antivirals and reduced prescribing of corticosteroids and antibiotics, also have potential to reduce the COI and improve outcomes. There is also growing support for various non antiviral generic drugs including statins, which may reduce mortality from severe influenza through mediation of the immune response and are widely available at low cost [31-33].

Sensitivity analysis confirms the cost-of-illness for HPAI falls dramatically to 4% of the average if hospital care and the associated diagnostic testing and pharmaceuticals are excluded. In this scenario the majority (86.6%) of costs are related to outpatient medicines. The absence of hospital care for patients suffering H5N1 is a likely scenario considering the limited health service resources available in Cambodia and would undoubtedly be associated with high rates of mortality. Conversely the maximum care afforded by hospital admittance, whilst undoubtedly reducing mortality, may result in an almost three-fold increase in the COI, with the cost of intensive care hospitalisation and pharmaceuticals being major contributors.

Our research did not identify the payer of the costs associated with illness and it is unknown how much of the medical costs were out-of-pocket expenses for the patients and their families, as compared to that subsidised by the government, non-government organisations, insurance or health equity funding. The patient or family expenses $44.82 (or $55.77 including outpatient medical costs) are considerable when they are considered with respect to the average income of $237 per month and prohibitive when compared to the average self-employed income gained in agriculture of $61 per month [20]. Additionally these approximates likely underestimate the cost of illness to a family as they may not include all over-the-counter medications utilised, loss of income related to care in the home, costs incurred for paying others to fulfil labour commitments and other costs incurred during hospitalisation, such as user charges for services provided. Indeed it has been estimated that two-thirds of total health expenditure in Cambodia is from patients’ out-of-pocket spending at the time of care, mainly for self-medication and private services [34]. As in other countries, catastrophic health expenditure has been identified as a major cause of indebtedness and destitution among the rural poor [34]. Access to free, quality, tertiary hospital care at a provincial level will be important to improving health care outcomes and reducing the burden of HPAI.

Additional limitations of this study include: the use of charges in place of costs for pharmaceuticals and diagnostic tests which may overestimate the contribution of these resources to the COI; and the lack of resource costs for patients who travelled to Vietnam. While this study represents all laboratory confirmed HPAI patients at the time of data collection, the number of cases is low and findings may differ from that during a serious pandemic scenario, when health care services are likely to be overwhelmed and incomes threatened. The true extent of disease attributable to H5N1 in Cambodia is also likely to be substantially higher than that described by the identified cases. The majority of HPAI infections in birds and humans occur in resource-poor areas where access to appropriate health care and formal diagnoses of H5N1 will be difficult to obtain. For these reasons it is probable that a significant proportion of those who contract the disease may go undetected, contributing to selection bias for our sample. Indeed a meta-analysis of studies, which assesses seroprevalence data for H5N1, reports a 1-2% infection rate in exposed populations [35]. The majority of mild or subclinical cases remain unidentified, and the number of deaths from H5N1 is also likely to be underestimated [35]. Thus, whilst the cases reported in this article likely represent the most severe infections with very poor clinical outcomes there will be a greater burden of disease resulting from HPAI, than that which has been captured by this data.

## Conclusion

Cases of highly pathogenic avian influenza have been characterised by young patient age, delays in hospitalisation, lack of adequate antiviral therapy, poor access to mechanical ventilation and a high fatality rate. Many patients died within a day of admission and may have progressed to a stage where treatment was unlikely to impact on clinical outcome. The substantial distances travelled to the admitting hospital and the costs associated with hospitalisation may have delayed patients and their caregivers from seeking appropriate treatment. Indeed the cost-of-illness for HPAI in Cambodia is relatively high, compared to other febrile illnesses, and varies considerably depending on the level of care received. Medical provider costs, particularly diagnostic testing and pharmaceutical costs were major contributors and reduction of such could substantially reduce the total cost of illness. Improved pharmaceutical management including the prompt initiation of antivirals may not only improve outcomes but also lower costs. Patient or family costs are considerable and will have a marked impact on poor families.

A highly pathogenic influenza outbreak in Cambodia has the potential to be a catastrophic event both from a loss of life and an economic perspective. Decision making for pandemic influenza preparedness should consider the potentially substantial cost of illness, both to the public health system and to the patient or patient’s family, and areas for improving patient care.

## Competing interests

The authors declare that they have no competing interests.

## Authors’ contributions

KHW contributed to the design of the study, analysis and interpretation of the data and manuscript preparation. TD contributed to the study conception, design and supervision and manuscript revisions. RC contributed to study conception and manuscript revisions. AH, ML, KB and ST contributed to the field collection of data and manuscript revisions. All authors read and approved the final manuscript.

## Pre-publication history

The pre-publication history for this paper can be accessed here:

http://www.biomedcentral.com/1471-2458/13/549/prepub
